# Correction: Bay et al. Functional Characterization of the Solute Carrier LAT-1 (SLC7A5/SLC3A2) in Human Brain Capillary Endothelial Cells with Rapid UPLC-MS/MS Quantification of Intracellular Isotopically Labelled L-Leucine. *Int. J. Mol. Sci.* 2022, *23*, 3637

**DOI:** 10.3390/ijms24055029

**Published:** 2023-03-06

**Authors:** Cindy Bay, Gzona Bajraktari-Sylejmani, Walter E. Haefeli, Jürgen Burhenne, Johanna Weiss, Max Sauter

**Affiliations:** Department of Clinical Pharmacology and Pharmacoepidemiology, Heidelberg University Hospital, Im Neuenheimer Feld 410, 69120 Heidelberg, Germany

The authors would like to make the following corrections to the publication [1]:

The authors accidentally mixed up the place of the numbers 2 and 3 in the designation “SLC3A2” of the heterodimeric partner of LAT-1, namely CD98 (4F2hc). Therefore, on various occasions throughout the paper, we incorrectly wrote the designation as “SLC2A3” (which is the designation of GLUT3) instead of its correct designation “SLC3A2”. Throughout the whole text, including the title, citation and Figure 5 legend, the authors would like to replace “SLC2A3” with “SLC3A2”.

Additionally, in Section 4.10, the cell line “hCMEC/D3” was misspelled as “HPMEC/D3”.

The authors apologize for any inconvenience caused and state that the scientific conclusions are unaffected. This correction was approved by the Academic Editor. The original publication has also been updated.
