# Supplementary material for: Functional Characterization of the Solute Carrier LAT-1 (SLC7A5/SLC2A3) in Human Brain Capillary Endothelial Cells with Rapid UPLC-MS/MS Quantification of Intracellular Isotopically Labelled L-Leucine
Source: Int J Mol Sci. 2022 Mar 26;23(7):3637. doi: 10.3390/ijms23073637 (PMC8998838; doi:10.3390/ijms23073637)
Supplement: Supplementary file 1 [file ijms-23-03637-s001.zip › ijms-1600183-supplementary.pdf]

Functional characterization of the solute carrier LAT-1 (SLC7A5/SLC2A3) in human brain capillary endothelial cells with rapid UPLC-MS/MS quantification of intracellular isotopically labelled L-leucine

Cindy Bay[1], Gzona Bajraktari-Sylejmani[1], Walter E. Haefeli, Jürgen Burhenne, Johanna Weiss, Max Sauter*

Department of Clinical Pharmacology and Pharmacoepidemiology, Heidelberg University Hospital, Im Neuenheimer Feld 410, 69120, Heidelberg, Germany

[1]These authors contributed equally to this work.

*Correspondence: max.sauter@med.uni-heidelberg.de

Supplementary material

Table S1: Calibration curves of the validation batches, calculated with linear regression and $1/x^2$ weighting.

Validation batch	R^2	Equation
#1	0.9980	y = 0.01740 x + 0.00011
#2	0.9948	y = 0.01765 x + 0.00021
#3	0.9956	y = 0.01630 x + 0.00037

Table S2: Accuracy and precision data for the 24 h autosampler stability determination.

QC level	Accuracy [%]	Precision [% CV]
Low QC	100.3	7.87
Mid QC	107.0	1.02
High QC	98.8	8.94

N = 3 replicates at each QC concentration.

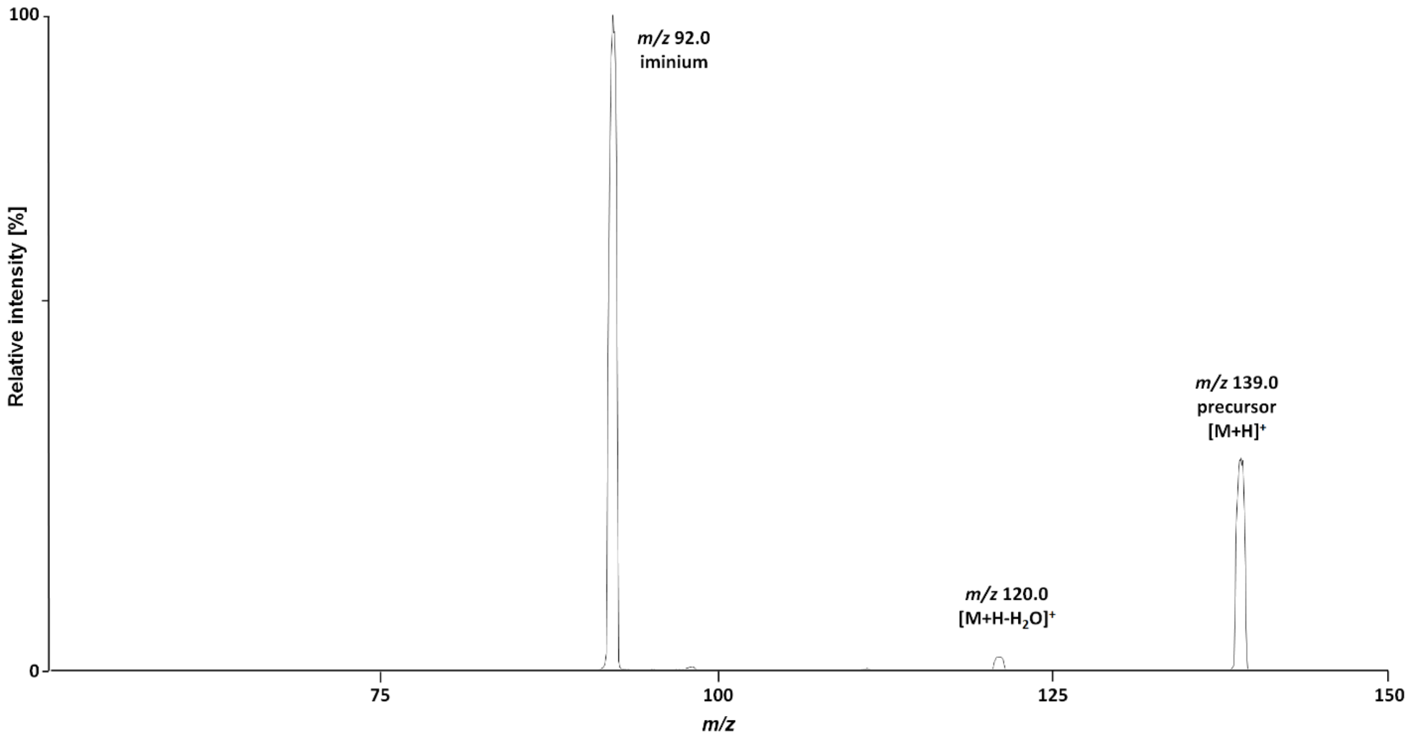

Figure S1. Product ion spectrum of [$^{13}C_6$, ^{15}N]-L-leucine at the optimal collision energy of 16 V for the second observed product ion at *m/z* 120.0. However, the iminium ion fragment is still substantially more intense, demonstrating its superior sensitivity for selected reaction monitoring.

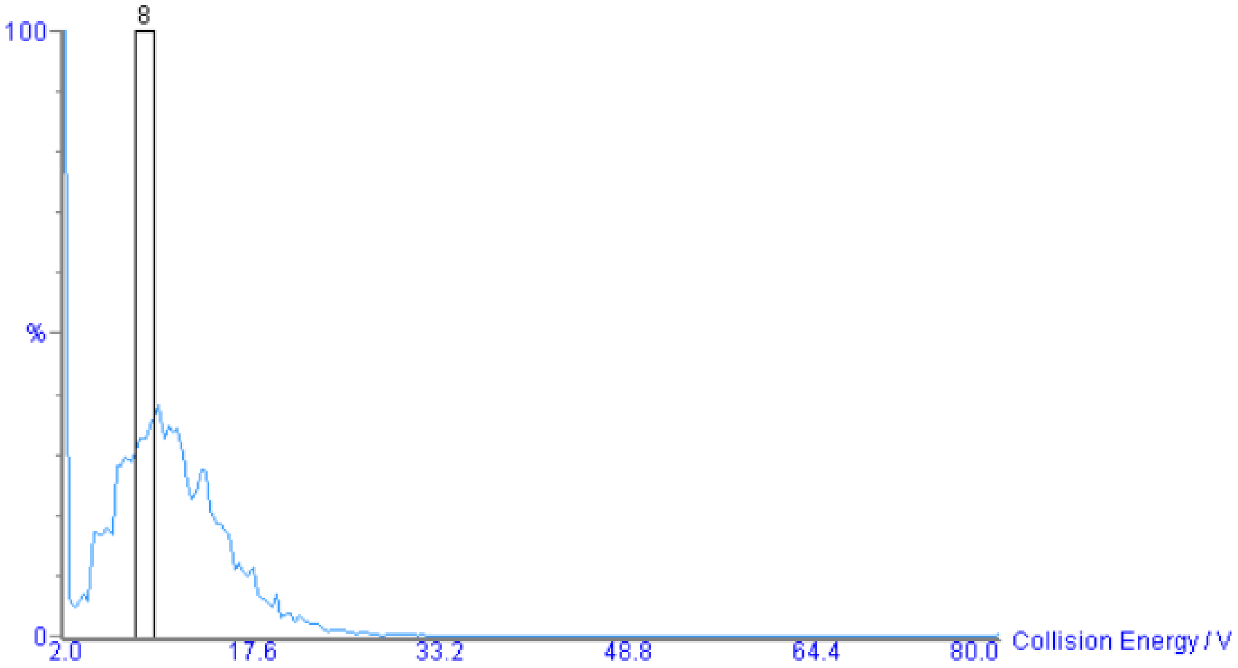

Figure S2. Optimization of the collision energy for the monitoring of the product iminium ion. The highest intensity for the corresponding transition of *m/z* 139.0 → 92.0 was achieved at a collision energy of 9 V.

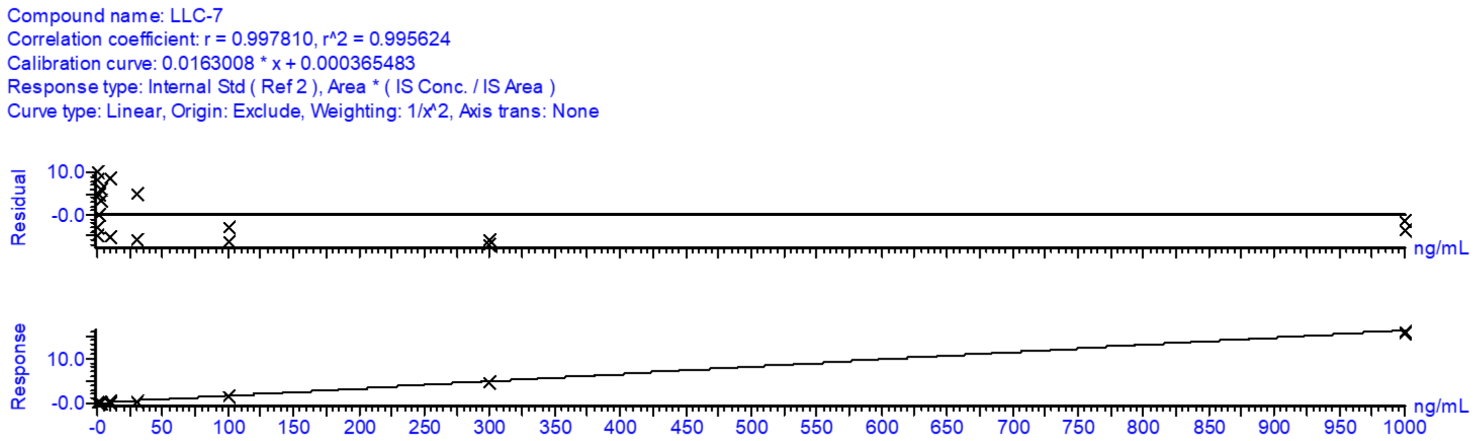

Figure S3. Exemplary calibration curve with residuals of validation batch #3.
